# Equity of access to primary healthcare for vulnerable populations: the IMPACT international online survey of innovations

**DOI:** 10.1186/s12939-016-0351-7

**Published:** 2016-04-12

**Authors:** Lauralie Richard, John Furler, Konstancja Densley, Jeannie Haggerty, Grant Russell, Jean-Frederic Levesque, Jane Gunn

**Affiliations:** Primary Care Research Unit, Department of General Practice, Faculty of Medicine, Dentistry and Health Sciences, University of Melbourne, 200, Berkeley street, Melbourne, VIC 3004 Australia; St. Mary’s Research Centre, 3830 Avenue Lacombe, Hayes Pavilion, suite 4720, Montreal, Qc H3T 1M5 Canada; School of Primary Health Care, Monash University, Building 1, 270 Ferntree Gully Road, Notting Hill, VIC 3168 Australia; Bureau of Heath Information, Level 11, Sage Building, 67 Albert Avenue, Chatswood, NSW 2067 Australia; Centre for Primary Health Care and Equity, UNSW, Sydney, 2052 Australia

**Keywords:** Access, Primary healthcare, Vulnerable populations, Innovations, Environmental scan, Online survey

## Abstract

**Background:**

Improving access to primary healthcare (PHC) for vulnerable populations is important for achieving health equity, yet this remains challenging. Evidence of effective interventions is rather limited and fragmented. We need to identify innovative ways to improve access to PHC for vulnerable populations, and to clarify which elements of health systems, organisations or services (supply-side dimensions of access) and abilities of patients or populations (demand-side dimensions of access) need to be strengthened to achieve transformative change. The work reported here was conducted as part of IMPACT (Innovative Models Promoting Access-to-Care Transformation), a 5-year Canadian-Australian research program aiming to identify, implement and trial best practice interventions to improve access to PHC for vulnerable populations. We undertook an environmental scan as a broad screening approach to identify the breadth of current innovations from the field.

**Methods:**

We distributed a brief online survey to an international audience of PHC researchers, practitioners, policy makers and stakeholders using a combined email and social media approach. Respondents were invited to describe a program, service, approach or model of care that they considered innovative in helping vulnerable populations to get access to PHC. We used descriptive statistics to characterise the innovations and conducted a qualitative framework analysis to further examine the text describing each innovation.

**Results:**

Seven hundred forty-four responses were recorded over a 6-week period. 240 unique examples of innovations originating from 14 countries were described, the majority from Canada and Australia. Most interventions targeted a diversity of population groups, were government funded and delivered in a community health, General Practice or outreach clinic setting. Interventions were mainly focused on the health sector and directed at organisational and/or system level determinants of access (supply-side). Few innovations were developed to enhance patients’ or populations’ abilities to access services (demand-side), and rarely did initiatives target both supply- and demand-side determinants of access.

**Conclusions:**

A wide range of innovations improving access to PHC were identified. The access framework was useful in uncovering the disparity between supply- and demand-side dimensions and pinpointing areas which could benefit from further attention to close the equity gap for vulnerable populations in accessing PHC services that correspond to their needs.

**Electronic supplementary material:**

The online version of this article (doi:10.1186/s12939-016-0351-7) contains supplementary material, which is available to authorized users.

## Background

A strong primary healthcare (PHC) system is paramount to optimising population health, yet PHC services are not always readily accessible [1, 2]. Striking differences in health still exist within and between populations, and inequities in access to PHC persist and tend to affect the most vulnerable^1^^a^ people in our communities, those with the most complex healthcare needs [3–5]. This was famously captured in the Inverse Care Law [6] which suggests that those with the greatest need often have, paradoxically, the poorest utilisation of healthcare services. From a human rights perspective, access to healthcare should be within reach of all, regardless of race, gender, culture, religion, political belief or socioeconomic condition [7]. Inextricably linked with access to healthcare is the notion of equity, which gives emphasis to its underpinning values of fairness and social justice [8–10].

Improving access to PHC has been on the global agenda for decades. It has been integrated as a central component to many contemporary health agreements (e.g. [11, 12]) and translated into substantial health service reforms internationally. Notwithstanding these efforts, there remains little evidence of equity of access to PHC at a population level. Furthermore, interventions designed to improve access to PHC for vulnerable populations are often highly fragmented and under-resourced [13–19]. Inequitable access to healthcare translates into unmet healthcare needs, worse and inequitable health outcomes and increased healthcare costs [20–22].

Equity of access to PHC is a major social determinant of health and is considered as a strategy for addressing health inequity [23]. The PHC sector as a whole has a responsibility to promote health equity as part of its social mandate [1]. This means developing interventions which support access via fair arrangements based on equal access to healthcare for all in equal need. Determinants of access to healthcare are amenable to change, both at a system level (e.g. transforming the way that health systems and organisations function; supporting the development of new professional roles and expanded scope of practice) and at an individual or population level (e.g. empowering patients to participate in decision-making processes regarding their care; advocating for community-led services). However, we are still striving to find effective ways of reaching equity of access to PHC to support those most in need, and to identify which aspects of services and abilities of people to strengthen in order to achieve transformative change.

The literature on access to healthcare is abundant, diverse and complex, offering varying definitions and conceptualisations [24–30]. In general, access can be defined as the opportunity or ease with which consumers or communities are able to use appropriate services in proportion to their need. In the past, it has been characterised with an emphasis on either attributes of health systems, organisations, services and providers (supply-side determinants of access) or abilities of individuals and populations to access services (demand-side determinants of access). More recently, a framework has been proposed that integrates both supply- and demand-side determinants in an attempt to capture the complexity of the phenomenon in the context of healthcare systems in perpetual transformation [24]. In line with an equity perspective [5, 31–34], conceptual frameworks of access should direct attention to demographic, social, economic, geographic and cultural factors which may structure the experience and opportunities of different social groups to reach and obtain appropriate healthcare [24, 31, 33, 34].

IMPACT (Innovative Models Promoting Access-to-Care Transformation) is a 5-year research program which brings together researchers in PHC, health services research and implementation science together with communities of practice in six regions in Canada and Australia. The aims of the research are to identify, refine and then trial best practice innovations to assist access to PHC, particularly for vulnerable populations. Here we report on the findings of an international online survey (referred to here as an environmental scan) of innovative interventions reported as enhancing access to PHC for vulnerable populations, undertaken as part of *IMPACT Project 1 – Scoping the innovations*. Our aim was to identify the breadth of innovations from the field. Environmental scanning is a research approach that uses wide-scope screening methods to identify the new, the unexpected and the emerging interventions, issues and challenges in health [35]. This study was developed as complementary to a scoping review of the published literature on access interventions, conducted independently of the environmental scan. Our rationale was that innovations may exist at the local, state and national levels while remaining undocumented in the literature.

## Methods

### Survey design

A brief 5-min online survey (see Additional file 1) was developed and hosted on *Qualtrics*, an online survey design software, which was selected for its ease of use and the quality of its user interface. A collaborative approach was chosen to design the survey. The IMPACT research team members were invited to participate in drafting the survey introduction and survey questions, as well as discussing the preferable structure of the survey (e.g. number of sections, item format). This led to the development of an initial version of the survey, which was piloted within the research team. Comments and suggestions provided by the team helped to improve the survey before it was piloted more broadly within the Department of General Practice at the University of Melbourne (Australia), in order to further address any usability and design issues before the official survey launch. The survey involved participants identifying and describing, from their own experience, an example of a program, service, approach or model of care that they considered innovative in helping vulnerable populations to access PHC services that meet their needs. Respondents were invited to identify the most striking components or aspects of the innovation. Information details gathered about innovations were as followed: name of innovation, geographic location, setting in which it was delivered, population group(s) targeted, core activities and processes, description of its innovative aspects, source(s) of funding. There was also a section of the survey enquiring about how we could learn more about the innovation, including options for the respondents to add a link to a website, a report or any other documentation that they would consider relevant. Different options were also offered to respondents in terms of language preference (the survey was available in English and French) and item format to complete the survey, the latter comprised of a mix of multiple choice options combined with description boxes, for those respondents who wanted to provide us with more detailed information. The survey, kept short and precise to optimise participation, also included a section about demographics of respondents.

### Planning survey dissemination

An iterative process involving the research team members and their networks was used to assemble an extensive email database of key PHC contacts, which included informants from PHC organisations, associations and university departments from Australia, Canada, UK and USA. Email contacts were then transferred into *Qualtrics* and email templates and schedules were created to facilitate survey dissemination (1^st^ email to raise awareness; 2^nd^ email initial contact with survey link, 3^rd^ email 2-week reminder, 4^th^ email final reminder). A comprehensive Google search was also undertaken to identify relevant online and social media channels to promote the survey. A Twitter account was created to build a list of followers and relevant Twitter accounts to follow as part of the social media campaign, in preparation for the survey launch. The social media campaign used Buffer, a social media management tool (www.buffer.com), which helped coordinate pre-programmed messages via Twitter and advertisements on other online platforms such as Linkedin and Facebook to build momentum around the study, ensure a high presence on social media and maximise response rate.

### Survey dissemination

The survey was disseminated widely amongst an international audience of PHC leaders, researchers, practitioners, policy makers and stakeholders using this combined email and social media approach. We also used a snowballing approach where the survey link would be shared within PHC networks and survey tweets would be commented on, liked and retweeted by a handful of followers, and then further shared by other people in extended networks, therefore increasing reach and visibility. The survey remained open for a 6-week period, from July 10^th^ to August 21^st^ 2014.

### Inclusion criteria

We deliberately decided not to use a predetermined definition of “innovation” so that we could identify, from the respondents’ perspectives, initiatives that they considered had made a difference in helping people to achieve improved access to services. Our focus was on trying to collect information from people who might have experienced a wide range of potentially innovative interventions, from a user, design, delivery or evaluation perspective. We included all innovations which were PHC focussed, primarily aimed at improving access and targeting vulnerable populations in our analysis.

### Conceptual framework

**Fig. 1:**
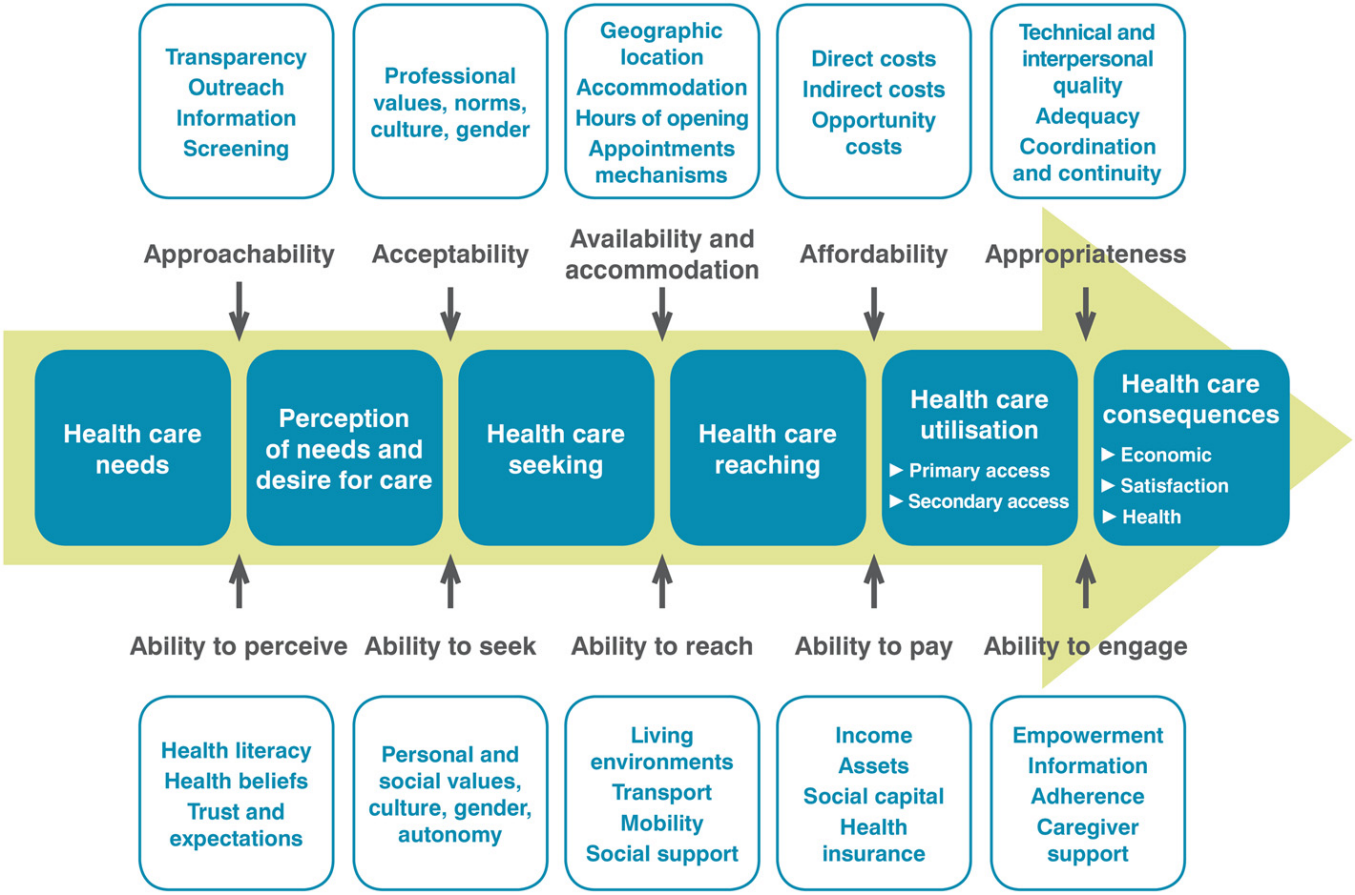
Conceptualisation of access adapted from Levesque et al. [24]

The Levesque et al. [24] access framework was used as the conceptual foundation for the study. The framework builds on previous conceptualisations of access (e.g. [25, 27, 30, 36–38], and is in continuous development with proposals which take into account social and health dimensions of access within an equity perspective [32]. Building on a comprehensive view of access articulated around factors pertaining to the healthcare system, individuals and context, the authors integrate both supply- and demand-side dimensions into their access framework, allowing operationalisation of access along the pathway of utilisation of care from perception of need through to the outcomes of service use. The framework is comprised of five dimensions of accessibility of care (approachability, acceptability, availability and accommodation, affordability, appropriateness) and five corresponding abilities of patients and populations to access care (ability to perceive, ability to seek, ability to reach, ability to pay, ability to engage) (Fig. 1). These dimensions of access are considered as interdependent constructs. The framework is arranged in pairs: each supply-side dimension of accessibility of care is mirrored by a matching demand-side ability of patients or populations to access services. The combination of a corresponding supply- and demand-side dimension is referred to here as “paired dimensions”. Operational definitions of each access dimension are described in Table 1.

**Table 1:** Definitions of access dimensions based on Levesque et a.l [24]

Supply-side dimensions of accessibility of services	Definitions	Demand-side abilities of patients to access services	Definitions
Approachability	Approachability of services relates to the fact that people facing healthcare needs can identify that some form of services exists, can be reached, and have an impact on their health.	Ability to perceive	Ability to perceive translates into the ability of people to identify their needs for care.
Acceptability	Acceptability of services relates to social and cultural factors determining the possibility for people to accept the aspects of a service.	Ability to seek	Ability to seek healthcare relates to factors that would determine expressing the intention to obtain healthcare.
Availability and accommodation	Availability and accommodation refers to the fact that health services (either the physical space or those working in healthcare roles) can be reached both physically and in a timely manner.	Ability to reach	Ability to reach healthcare relates to factors that would enable one person to physically reach service providers.
Affordability	Affordability reflects the economic capacity for people to spend resources and time to use appropriate services.	Ability to pay	Ability to pay for healthcare is described as the capacity to generate economic resources to pay for healthcare services without catastrophic expenditure of resources required for basic necessities.
Appropriateness	Appropriateness denotes the fit between services and clients' needs, its timeliness, the amount of care spent in assessing health problems and determining the correct treatment and the technical and interpersonal quality of the services provided.	Ability to engage	Ability to engage in healthcare relates to the participation and involvement of the client in decision-making and treatment decisions, which is in turn strongly determined by capacity and motivation to participate in care and commit to its completion.

### Analysis

We conducted a framework analysis [39] based on the Levesque et al. [24] access framework to all included innovations. Written descriptions of innovations were mapped against the framework to identify which access dimensions were addressed. An innovation could address more than one dimension of the access framework. Each description of innovation was specifically examined to see if it addresses supply-side only, demand-side only, or paired dimensions of access. All data were double coded (LR and alternately JG, JF). Quotes from each description of innovation were used to support the coding process, and these were referred to when discrepancies arose between coders in order to reach agreement. The innovations were also assessed in terms of their implementation level – micro (local or practice level), meso (state or regional level), and macro (national level) – and in terms of whether they involved the participation of the health sector, the social sector or both sectors (i.e. multisectoral initiatives). Finally, components of interventions were identified inductively through the analysis process to further exemplify each dimension of access. Descriptive statistics were used to characterise the innovations according to: country of innovation, sectors involved, population group(s) targeted, setting(s) in which the innovation is delivered, implementation level, funding sources and access dimensions addressed. All statistical analyses were conducted using STATA version 12 [40].

This study received full ethics approval from the University of Melbourne Human Research Ethics Committee (1442125.1). Participation in the survey was voluntary and consent was obtained by respondents filling out and submitting their survey. Plain Language Statements (English and French) were linked to the first screen of the online survey and attached to the email invitations for easy access.

## Results

**Fig. 2:**
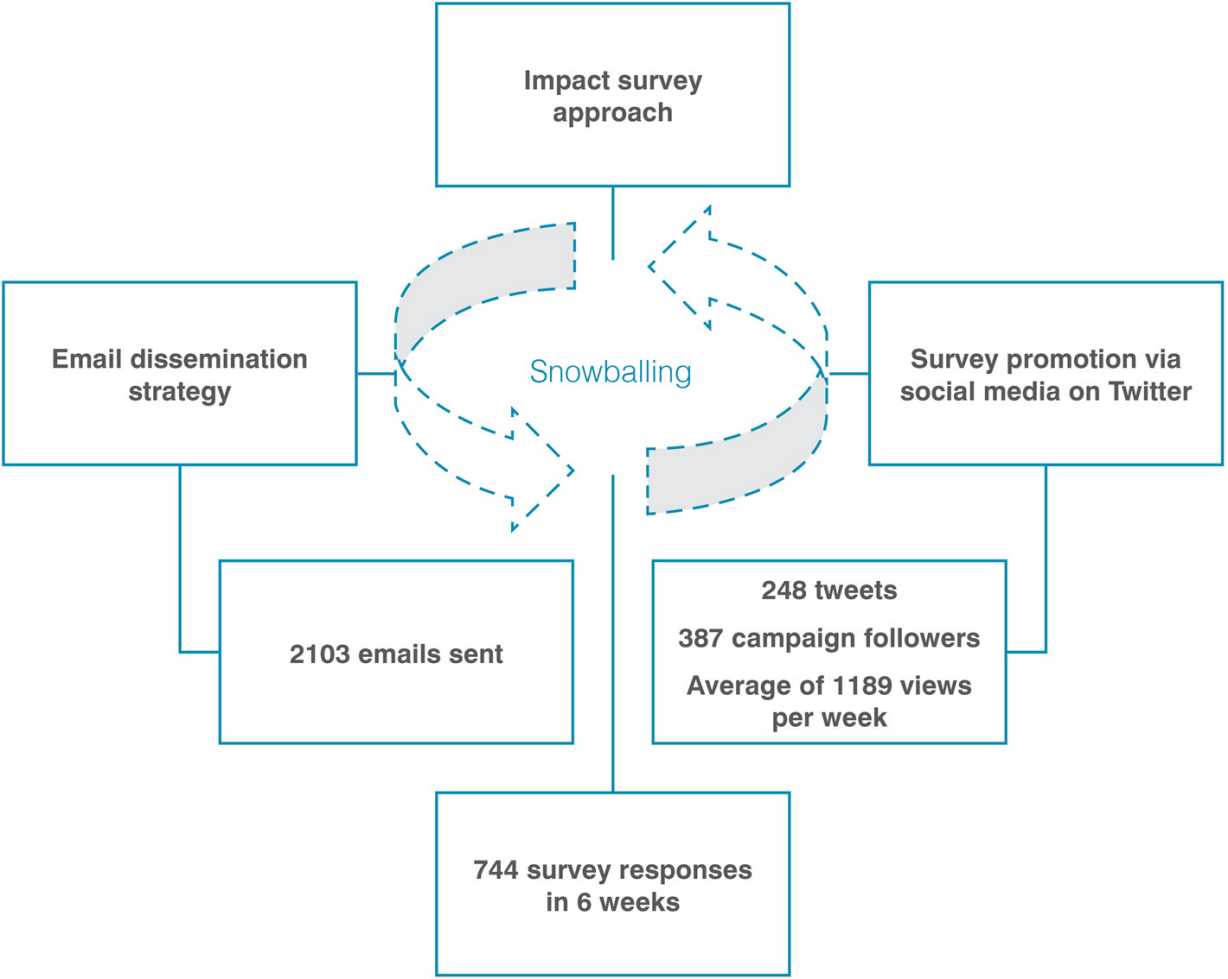
Survey procedure

**Fig. 3:**
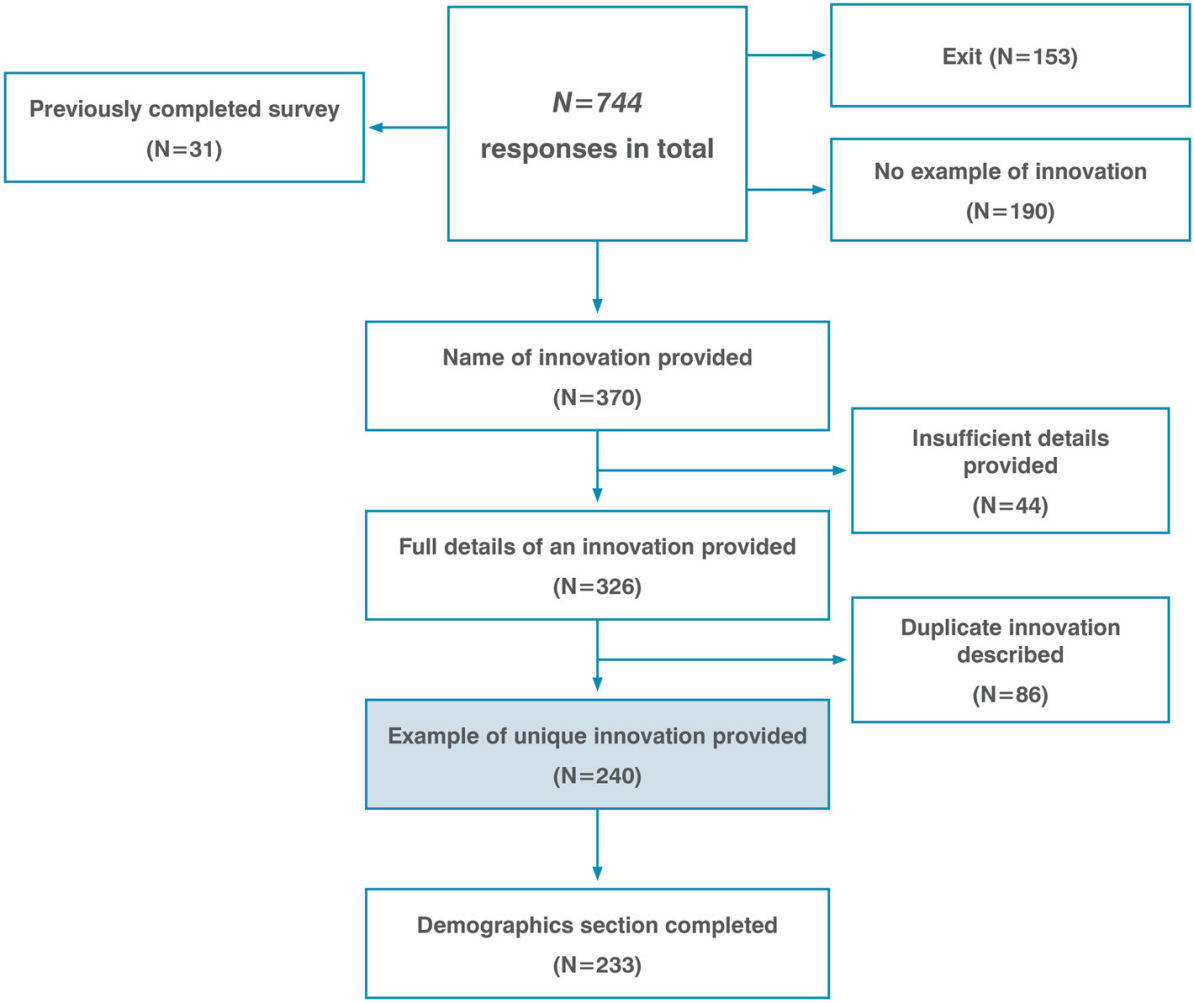
Survey results flowchart

Over 2000 emails were sent to key PHC informants and 248 tweets were posted on Twitter, creating a social media presence aimed at building interest in the survey topic. The social media campaign attracted 387 followers and the tweets were viewed 1189 times per week on average (Fig. 2). We recorded 744 survey responses over a 6-week period, describing 240 unique examples of innovations (Fig. 3).

**Fig. 4:**
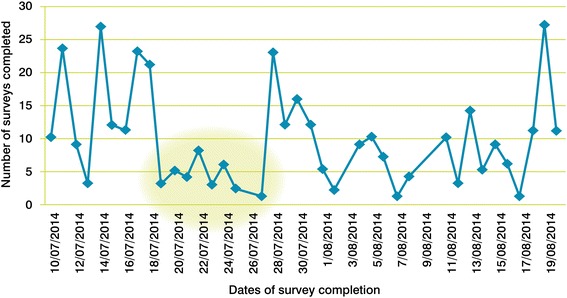
Number of surveys completed across the survey completion period

The number of surveys completed correlated to the dates that email invitations and reminders were sent (Fig. 4). Following the lull between July 20^th^ and 26^th^, we modified our social media campaign strategy in varying the content of our tweets, sharing papers of interest and emerging findings to encourage completion of the survey.

The survey was mainly completed in English (89.2 %). From the respondents who provided an example of innovation (name and description; *N* = 326), 233 (71.5 %) completed the demographics section. Most respondents were from Canada (47.6 %) and Australia (42.9 %). The majority of respondents were female (71.7 %), aged between 35 and 54 years old (48.5 %), had completed a postgraduate degree (72.1 %), worked as a researcher (32.2 %), general practitioner (25.3 %), nurse (24.9 %), or as a manager of PHC services (18.5 %), and reported an initiative that they either designed, implemented or evaluated (47.3 %), delivered as part of a program or service (45.1 %), or used themselves (11.8 %). The demographics of the survey respondents are presented in Table 2.

**Table 2:** Demographics of survey respondents (*N* = 233)

	Number	Percent
Respondents Country		
Canada	111	47.6 %
Australia	100	42.9 %
Other^a^	22	9.4 %
Respondents gender		
Female	167	71.7 %
Male	64	27.5 %
Rather not say	2	0.9 %
Respondents age		
18–25	11	4.7 %
26–34	35	15.0 %
35–54	113	48.5 %
55–64	62	26.6 %
65 or over	12	5.2 %
Respondents qualification		
Certificate/diploma	15	6.4 %
Postgraduate degree	168	72.1 %
Secondary school/High school	1	0.4 %
Undergraduate degree	49	21.0 %
Primary area of work		
Researcher	75	32.2 %
General Practitioner	59	25.3 %
Nurse	58	24.9 %
Manager PHC	43	18.5 %
Other health provider	20	8.6 %
Student	20	8.6 %
Government	11	4.7 %
Volunteer worker	8	3.4 %
Educational role	7	3.0 %
Not in paid work	6	2.6 %
Social worker	4	1.7 %
Other	19	8.2 %
How respondents know about the innovation		
Know program because designed it	112	47.3 %
Know program because delivered it	107	45.1 %
Know program from colleague	40	16.9 %
Know program from using it	28	11.8 %
Know someone who used program	23	9.7 %
Know program Internet/Media	9	3.8 %
Know program - Other	28	11.8 %

^a^Cameroon, India, Indonesia, Ireland, Israel, Italy, Netherlands, New Zealand, Sudan, Switzerland, United Kingdom of Great Britain/Northern Ireland, United States of America

### Characteristics of innovations

The general characteristics of the innovations are shown in Table 3. As no major difference was found by country of origin, we have combined the findings from respondents from different countries.

**Table 3:** General characteristics of innovations (*N* = 240)

	Number	Percent
Country of innovations^a^		
Canada	108	45.0 %
Australia	98	40.8 %
Other	34	14.2 %
Sectors involved		
Health	171	71.3 %
Social	1	0.4 %
Both	68	28.3 %
Population groups targeted^b^		
Low income individuals/families	70	35.0 %
People living with a chronic disease	66	33.0 %
Homeless people	56	28.0 %
Indigenous	55	27.5 %
People living with a mental health illness	52	26.0 %
Refugees	43	21.5 %
Culturally And Linguistically Diverse communities	34	17.0 %
Drug users	34	17.0 %
Elderly	34	17.0 %
Children/Adolescents	32	16.0 %
People with disability	24	12.0 %
Victims of violence/abuse	21	10.5 %
Lesbian Gay Bisexual Transgender Intersex	15	7.5 %
Pregnant women/maternal health	11	5.5 %
Remote/rural communities	9	4.5 %
No particular population group targeted	26	10.8 %
Multiple population groups targeted	102	51.0 %
Other	20	10.0 %
Settings where innovations are delivered^b^		
Setting Community Health Centre	122	50.8 %
Setting General Practice/Family Medicine Group	96	40.0 %
Setting Mobile clinic/Outreach	70	29.2 %
Setting at the Home	64	26.7 %
Setting NGO	50	20.8 %
Setting Telephone	43	17.9 %
Setting Hospital	41	17.1 %
Setting Online	21	8.8 %
Setting School/educational facility	10	4.2 %
Setting Shelter	8	3.3 %
Setting Other	47	19.6 %
Innovation delivered in multiple settings	137	57.1 %
Implementation level		
Micro	217	90.4 %
Meso	17	7.1 %
Macro	6	2.5 %
Sources of funding^b^		
Financed by Government	182	76.8 %
Financed by Non-for-profit	72	30.4 %
Financed by Private sector	21	8.9 %
Financed - I don’t know	19	8.0 %
Financed by User payment	12	5.1 %
Financed - by other	26	11.0 %
Number of funding sources involved		
1	163	68.8 %
2	58	24.5 %
3	11	4.6 %
4	5	2.1 %

^a^Cameroon, India, Indonesia, Ireland, Israel, Italy, Netherlands, New Zealand, Sudan, Switzerland, United Kingdom of Great Britain/Northern Ireland, United States of America

^b^Multiple responses allowed for this question

#### Country of innovations

The innovations reported in the survey originated from 14 countries, with the majority coming from Canada (45.0 %) and Australia (40.8 %).

#### Sectors involved

Innovations were primarily health sector focused (71.3 %), with only 28.3 % of them involving both health and social sectors.

#### Population groups

More than half of the innovations were directed at multiple vulnerable population groups (51.0 %), of which low income individuals and families (35.0 %), people living with a chronic disease (33.0 %), homeless individuals (28.0 %) and indigenous communities (27.5 %) were most frequently targeted. Approximately 10.0 % of innovations did not focus on a particular population group.

#### Settings

Around half (50.8 %) the innovations identified were delivered in the community health setting, 40.0 % in the General Practice or Family Medicine Group setting, and 29.2 % in the mobile/outreach clinic setting. The majority of innovations were delivered in multiple settings (57.1 %).

#### Implementation level

Almost all innovations were operating at the practice or community level (90.4 %). Only 7.1 % were implemented at a regional level (state, province) and 2.5 % at a national level.

#### Funding sources

Most initiatives reported receiving government funding (76.8 %) and around one third reported receiving funding from non-governmental organisations (NGOs) (30.4 %). Innovations were mainly reported as being funded by a single source (68.8 %).

#### Primary dimensions of access addressed

Detailed features of innovations relating to dimensions of access are presented in Table 4. Overall, the majority of innovations addressed dimensions of access pertaining to the supply-side only (72.9 %), a very small percentage of innovations solely addressed demand-side dimensions of access (0.8 %) and slightly more than a quarter of innovations (26.3 %) addressed both supply- and demand-side dimensions.

**Table 4:** Dimensions of access featured in the descriptions of innovations (*N* = 240)

	Number	Percent
Primary dimensions of access addressed		
Supply-side dimensions only	175	72.9 %
Demand-side dimensions only	2	0.8 %
Both	63	26.3 %
Supply-side dimensions of accessibility of services^a^		
Appropriateness	157	65.4 %
Approachability	134	55.8 %
Availability and accommodation	112	46.7 %
Acceptability	40	16.7 %
Affordability	29	12.1 %
Number of supply-side dimensions per innovation		
0	2	0.8 %
1	88	36.7 %
2	76	31.7 %
3	64	26.7 %
4	10	4.2 %
Demand-side abilities of patients/populations to access services^a^		
Ability to engage	47	19.6 %
Ability to perceive	24	10.0 %
Ability to seek	23	9.6 %
Ability to reach	6	2.5 %
Ability to pay	6	2.5 %
Number of demand-side dimensions per innovation		
0	175	72.9 %
1	36	15.0 %
2	18	7.5 %
3	10	4.2 %
4	1	0.4 %
Overall number of dimensions of access targeted (supply- and demand-side combined)		
1	66	27.5 %
2	70	29.2 %
3	67	27.9 %
4	22	9.2 %
5	10	4.2 %
6	3	1.3 %
7	1	0.4 %
8	1	0.4 %
Paired dimensions of access^b^		
Appropriateness & Ability to engage	33	13.8 %
Approachability & Ability to perceive	21	8.8 %
Acceptability & Ability to seek	6	2.5 %
Availability & Ability to reach	4	1.7 %
Affordability & Ability to pay	0	0.0 %
Number of pairs per innovation		
0	187	77.9 %
1	45	18.8 %
2	6	2.5 %
3	1	0.4 %
4	1	0.4 %

^a^An innovation could address more than one access dimension. Therefore, the number of innovations does not totalise 240 for this section of the table

^b^An innovation could address more than one pair of access dimension. Therefore, the number of innovations does not totalise 240 for this section of the table

#### Supply-side dimensions of access

When looking specifically at the supply-side dimensions of accessibility of services, appropriateness (65.4 %), approachability (55.8 %), and availability and accommodation (46.7 %) appeared as the most commonly addressed dimensions reported in the descriptions of innovations. The majority of innovations (95.0 %) addressed between 1 and 3 supply-side dimensions.

#### Demand-side dimensions of access

Most descriptions provided by the respondents did not feature demand-side abilities of patients or populations to access services (72.9 %). When identifiable in the descriptions of innovations, the most frequently reported demand-side dimension was ability to engage (19.6 %), followed by ability to perceive (10.0 %) and ability to seek (9.6 %).

#### Paired dimensions

Most innovations did not target paired supply- and demand-side dimensions of access (77.9 %) - dimensions of accessibility of services were generally not combined with their corresponding abilities of patients/populations to access services. When considering both supply- and demand-side dimensions together, most innovations addressed 3 dimensions or less (84.6 %). Only one fifth of innovations (18.8 %) targeted 1 pair, and less than 3 % of innovations targeted 2 pairs or more. The most common pairs were appropriateness/ability to engage (13.8 %) and approachability/ability to perceive (8.8 %).

#### Population groups and settings in relation to specific access dimensions

When looking at the target population groups in relation to the dimensions of access addressed in the reported initiatives, the findings reveal how specific dimensions may relate to the needs of specific groups. For example, initiatives addressing approachability of services targeted vulnerable, marginalised and culturally diverse groups (e.g. homeless people, low income individuals and families, indigenous communities and refugees) for which identifying what services exist that correspond to their needs might be a challenge. In particular, refugees were a predominant population group targeted in interventions addressing the ability of people to perceive the need for care, with components of interventions focusing on health literacy and education to increase knowledge about health and health systems. When looking at initiatives addressing acceptability of services, Indigenous people were the predominant population group targeted, with interventions giving emphasis to cultural factors determining the acceptability of healthcare services according to their own values, beliefs and norms. People living with a chronic disease were the predominant group in interventions trying to enhance the ability to seek and engage in healthcare services, with components of interventions focusing on enhancing patients’ autonomy or aimed at facilitating the participation of patients in their process of care. Populations with complex healthcare needs require interventions which rely on multidisciplinary approaches, integrated network of services and continuity of care processes, which were key components of interventions described as part of initiatives addressing appropriateness of services. No major difference was found in settings where interventions were delivered to address specific dimensions of access. Overall, the community health setting was predominant, alongside General Practices, mobile clinics/outreach and NGOs almost evenly distributed across all dimensions. The most prevalent components of interventions identified in the survey to further exemplify each dimension of access (supply- and demand-side) are presented in Table 5. Illustrative vignettes were also assembled to represent the different types of innovations reported in the survey (Table 6).

**Table 5:** Components of interventions per access dimension

	Components of interventions relating to access dimensions				
Supply-side dimensions of accessibility of services	Approachability	Acceptability	Availability and Accommodation	Affordability	Appropriateness
Examples of components of interventions per dimension of access	Navigation and information	Adaptation to needs of specific populations	Outreach from PHC into community setting	Defraying costs to patients	Comprehensive PHC team - One Stop Shop
	Facilitated referral for services	Community health worker	Virtual consultation with health provider		PHC network with community organisations
	Proactive identification of needs (e.g. early health assessments)		Expanded scope of practice of health professionals		PHC Case Manager
	Transparency		Geographic location of PHC services		
Demand-side abilities of patients to access services	Ability to perceive	Ability to seek	Ability to reach	Ability to pay	Ability to engage
Examples of components of interventions per dimension of access	Health and service literacy	Education and self-management coaching (e.g. access to education material or devices to track your own health)	Transportation options to access services	No out-of-pocket costs for patients	Proactive role and participation of patients and carers (e.g. setting goals, priorities and actions for the healthcare plan)
		Peer-support workers	Connecting with social groups/social support		Community governance model (e.g. community-led services)

**Table 6:** Vignettes of the types of interventions

Illustrative vignettes of interventions			
Types of vignette	Description of intervention	Access dimension(s) addressed*	Components of interventions relating to access dimensions*
Innovations that illustrate the most targeted supply-side dimensions	Name of the innovation: PACER Model of Primary Mental Health Care Country of innovation: Australia Setting: mobile service Target population: people living with a mental illness What does it do? PACER is a mobile emergency mental health program that teams a Police officer with mental health training and an experienced mental health clinician to respond to mental health crises encountered by Police. This program offers improved coordination of activities between emergency services (Ambulance and Police) and the area of mental health services. The PACER team’s complementary skill sets ensure personal and community safety during the crises and skilled in-time assessment, treatment and referral as appropriate.	Approachability Acceptability Availability and accommodation Appropriateness	Mobile clinic Outreach from PHC into community setting Expanded hours Multi-sectoral network
	Name of the innovation: The Alex Community Health Bus Country of innovation: Canada Setting: mobile service Target population: low income individuals and homeless people What does it do? The Alex Community Health Bus is a mobile clinic providing healthcare services to low income individuals and homeless people five days a week. The Alex Health Bus stops at a number of locations on its weekly route, including low-income seniors housing complexes and homeless shelters. It provides healthcare and education services, and facilitates referrals to a wide range of PHC and community organisations. It is also a roaming food bank, with hampers and emergency food on-board for those in need.	Approachability Availability and accommodation Appropriateness	Mobile clinic Outreach from PHC into community setting Facilitated referral for services Multi-sectoral network
	Name of the innovation: Bromley By Bow Health Centre Country of innovation: UK Setting: community organisation Target population: no particular population group What does it do? Bromley By Bow Health Centre is a community organisation in East London, working in one of the most deprived neighbourhoods of the city. As a healthy living centre, it offers a wide range of health services such as consultations with general practitioners and psychologists, home visits, antenatal and baby clinics, family planning services, blood clinics, new patient health checks and nurse clinics.	Approachability Availability and accommodation Appropriateness	Geographic location of PHC services Comprehensive PHC team – One Stop Shop PHC network with community organisations
	Name of the innovation: Cool Aid Community Health Centre Country of innovation: Canada Setting: community health centre Target population: homeless people, people living with a mental illness or disability, low income individuals, people facing addiction problems What does it do? Cool Aid provides primary healthcare, counseling, dental care and a dispensing pharmacy. These services are provided by a multidisciplinary team and rely on a holistic approach to healthcare that includes a strong patient-centered vision, offering opportunities for patients to take part in their care and making decisions regarding their health. This team includes doctors and nurses, counselors, a nutritionist, a psychiatrist, an acupuncturist, a podiatrist, dentists and dental hygienists, a pharmacist and a pharmacy technician. In particular, Cool Aid offers shelter services, health and dental care, mental health and employment support, food supplies, community engagement programs, outreach clinics, peer-based support groups, harm reduction services, onsite pharmacy with opiate substitution program, and group medical visits for individuals with complex social, psychiatric and medical needs.	Approachability Acceptability Availability and accommodation Appropriateness	Comprehensive PHC team – One Stop Shop Adaptation to needs of specific subpopulation Outreach from PHC into community setting In-reach from specialised services to PHC Group visits Patient-centered care
	Name of the innovation: PRIME: A Health Centre for Seniors Country of innovation: Canada Setting: health centre and residence Target population: elderly people What does it do? PRIME: A Health Centre for Seniors offers a program aimed at keeping seniors healthy and living in their own homes. PRIME provides alternatives to entering a personal care home by offering an all-inclusive health service including medical care, personal care, socialisation and exercises, after hours support, rehabilitation, day program and home care coordination, amongst other services for seniors. Services are provided based on a collaborative, multidisciplinary approach. Transportation is provided to help people attend the service.	Approachability Availability and accommodation Appropriateness	Comprehensive PHC team – One Stop Shop Integrated healthcare network Outreach from PHC into community setting Transportation
Innovation that illustrates the most targeted demand-side dimensions	Name of the innovation: Byron Bay homeless breakfast Country of innovation: Australia Setting: community organisation Target population: homeless people What does it do? Byron Bay homeless breakfast is run once a week at the community centre by volunteers who provide free meals for the homeless and anyone in need in the neighbourhood. The local community health centre staff attend breakfast and use it as an opportunity to provide primary healthcare services to the people who come to breakfast.	Ability to perceive Ability to engage Approachability Acceptability Availability and accommodation	Adaptation to needs of specific subpopulation Outreach from PHC into community setting PHC network with community organisations
	Name of the innovation: MyGRiST Country of innovation: UK Setting: online Target population: people living with a mental illness What does it do? MyGRiST is an online tool designed to help people self-assess and manage risks and safety associated with their mental health problems, with the aim of promoting wellbeing. It is a companion tool to a suite of clinical tools that have been developed based on a model of clinical risk assessment founded on the most recent evidence in the field. MyGRiST collects identical information to the clinical tools, but using language and a format co-designed with mental health service users. This helps empower patients and enables them to tell their story and communicate risk information to clinicians. It does this by providing them with a script - i.e. an output report which clearly indicates where patients’ main concerns are. Reports can be shared online, for purposes of remote supervision. Patients' personalised output reports also contain self-management advice and planning.	Ability to perceive Ability to seek Ability to engage Approachability Acceptability Availability and accommodation Appropriateness	Virtual monitoring of health condition Self-management coaching Common tools
	Name of the innovation: Diabetes Coordination and Assessment Service Country of innovation: Australia Setting: phone-based, primary healthcare organisation Target population: people living with diabetes What does it do? Diabetes Coordination and Assessment Service is a phone-based care coordination service aiming to promote chronic disease self-management (diabetes in particular) through screening, triage, assessment, coaching, referral and follow-up. It assists primary healthcare to connect people with services that correspond to their needs. Services also include public diabetes groups, individual coaching sessions and specialist clinics.	Ability to seek Ability to engage Approachability Availability and accommodation Appropriateness	Self-management coaching Facilitated referral for services Proactive follow-ups Navigation and information Virtual consultation of health provider
	Name of the innovation: Living Well with COPD Country of innovation: Canada Setting: online Target population: people living with chronic obstructive pulmonary disease (COPD) and their family What does it do? Living Well with COPD is a self-management education program developed to help people living with COPD and their family to take charge and cope with their disease, in collaboration with their healthcare team. The goal is to facilitate the adoption of healthy lifestyle behaviors and the skills needed to ensure optimal management of COPD on a day-to-day basis. It provides free access to a large number of educational modules to help manage COPD and resources about how to navigate the healthcare system.	Ability to perceive Ability to seek Ability to engage Approachability Affordability Appropriateness	Self-management coaching Navigation and information Defraying costs to patients
Innovation that combines the most targeted pairs of access determinants	Name of the innovation: The HOME study Country of innovation: Australia Setting: home-based Target population: Aboriginal and Torres Strait Islander people with complex chronic disease What does it do? The Home based Outreach chronic disease Management Exploratory Study (HOMES) explores novel approaches to address chronic disease management in home-based outreach settings for Aboriginal and Torres Strait Islander people. The integrated family-based chronic disease management program involves the engagement and empowerment of families in the management and prevention of chronic disease; comprehensive needs assessment (family health, social situation and needs, physical healthcare needs and social and emotional wellbeing); and integration of health and health related care delivery to patients and their families to improve health outcomes.	Approachability-Ability to perceive Appropriateness-Ability to engage Ability to seek	Outreach from PHC into community setting System case manager Patient-centered care Advocacy Comprehensive PHC team – One Stop Shop
	Name of the innovation: IMAGINE Country of innovation: Canada Setting: community-based drop-in clinic Target population: marginalised and underserved communities What does it do? IMAGINE (Interprofessional Medical and Allied Groups for Improving Neighbourhood Environment) is an interprofessional, student-run community health initiative aimed at promoting and providing holistic healthcare to the core neighbourhoods of downtown Toronto. It offers outreach activities with community partners as well as health promotion educational workshops with clients.	Approachability – Ability to perceive Appropriateness – Ability to engage Ability to seek	Student-led services Comprehensive PHC team – One Stop Shop Outreach from PHC into community setting
	Name of the innovation: AMP (Access to Mental health in Primary care) Country of innovation: UK Setting: primary healthcare model implemented in different health and community settings Target population: people from underserved groups What does it do? The aim of the AMP Program is to increase access to high quality primary care mental health services for people from underserved groups. It provides services that are based on a patient-centered and culturally responsive approach. The AMP model is comprised of three core components: 1) community engagement; 2) primary care quality; 3) psychosocial interventions.	Approachability-Ability to perceive Appropriateness-Ability to engage	PHC network with community organisations Navigation and information Comprehensive PHC team – One Stop Shop PHC research embedded in continuous quality improvement Patient-centered care
	Name of the innovation: The Kalwun Development Corporation Country of innovation: Australia Setting: community health service Target population: Aboriginal and Torres Strait Islander people What does it do? The Kalwun Development Corporation provides services to Aboriginal and Torres Strait Islander people, based on a community controlled designed and led approach to the delivery of accessible, efficient, effective and appropriate comprehensive primary healthcare. The Kalwun Development Corporation offers a combination of primary healthcare and community-based services such as access to general practitioners, comprehensive screening, onsite allied health services, mobile outreach medical clinic on regular basis, immunisation and transport services. It also offers a program of care coordination to support patients with chronic diseases in accessing necessary services.	Appropriateness - Ability to engage Approachability Acceptability	Community governance model Adaptation to needs of specific subpopulation PHC network with community organisations Mobile clinic Transportation
Innovations that combine multiple access dimensions and bridge social and health sectors	Name of the innovation: Multicultural Health Brokers Country of innovation: Canada Setting: community organisation Target population: Immigrants, refugees/new comers What does it do? The Multicultural Health Brokers Co-operative supports families that are new to Canada to bridge between their own knowledge from their home country and Canada’s health, social services, education, justice, immigration and employment support systems. The Brokers are a group of 54 people who represent 22 different cultural and linguistic communities in Edmonton, Canada. They started as volunteers and were identified as natural leaders in their communities, and were brought in as a paid capacity with the Co-operative. The organisation offers a wide range of programs that cover social and healthcare needs.	Approachability Acceptability Availability and accommodation Appropriateness-Ability to engage	Community health worker Health service broker Adaptation to needs of specific subpopulation Outreach from PHC into community setting Navigation & information Advocacy
	Name of the innovation: Youth projects – The Living Room Primary Health Service Country of innovation: Australia Setting: primary health service, mobile/outreach Target population: homeless people What does it do? The Living Room is a Primary Health Service that provides free healthcare and support to improve the physical, mental and social wellbeing of individuals who are homeless or at risk of homelessness, disadvantaged or marginalised, with complex healthcare needs. It provides a wide range of services such as health and social assessments, professional nursing care, counselling and active support, first aid, medication management, and follow up to clients; housing support and referral; shower and laundry facilities; food and material aid; legal support; and art therapy. It uses an assertive outreach service model to respond to the after-hours healthcare needs of the homeless community, delivering services in public spaces and crisis accommodation.	Approachability Availability and accommodation Affordability Appropriateness	Comprehensive PHC team – One Stop Shop Adaptation to needs of specific subpopulation Mobile clinic
	Name of the innovation: Grameen PrimaCare Country of innovation: USA Setting: primary healthcare service Target population: immigrant women What does it do? Grameen PrimaCare is a non-profit organisation that provides underserved women from low-income immigrant communities with a high-quality, affordable primary healthcare and health promotion program, empowering them to lead healthier lives. Grameen PrimaCare is founded on a comprehensive approach to healthcare, providing its members with access to a broad range of primary healthcare services, a wellness centre, healthcare tools and a combination of discounted services. The practice is conveniently located to recruit women from different cultural backgrounds and its team is comprised of a bilingual female doctor, two nurse practitioners and one registered nurse. Also on staff is a team of 10–13 health coaches who work to motivate members to achieve health goals, implement customised care plans, coordinate care amongst providers and connect members to additional services and resources. Grameen PrimaCare offers support groups focusing on wellness education. The curriculum covers a range of topics that engage members in becoming active participants in their health. An online platform is also available and allows members to track their own health and access educational resources that support their journey towards better health and well-being.	Approachability-Ability to perceive Acceptability - Ability to seek Appropriateness-Ability to engage Affordability	Geographic location of PHC services Adaptation to needs of specific subpopulation Self-management coaching Virtual monitoring of health condition Comprehensive PHC team – One Stop Shop PHC network with community organisations
	Name of the innovation: The Blue Mountains Aboriginal healthy for life program Country of innovation: Australia Setting: partnership between community organisations, community health centres and General Practices Target population: Aboriginal and Torres Strait Islander people What does it do? The Blue Mountains Aboriginal healthy for life program is an Australian Government program that is aimed at helping Aboriginal and Torres Strait Islander people improve their health. Its specific objectives are to enhance the quality of life and health outcomes of Aboriginal and Torres Strait Islander people living with chronic and complex illnesses, and to reduce the incidence of such illnesses over time. The team is made up of two registered nurses, a male and female Aboriginal outreach worker, a Aboriginal child and family worker, a Healthy for life practice/project support officer and a program manager. The team assists by meeting in the family home or other preferred location to discuss health issues, providing a link to health professionals, doctors or specialists, and arranging regular health checks and transportation to health appointments.	Approachability Acceptability Appropriateness-Ability to engage	Facilitated referral for services Adaptation to needs of specific subpopulation Comprehensive PHC team – One Stop Shop PHC network with community organisations Transportation Community governance model

* Dimensions of access and components of interventions identifiable through the description of innovation provided by survey respondents

## Discussion

### Globally shared challenges

This environmental scan identified, in a timely and cost-effective manner, a wide-range of potentially promising innovations for improving access to PHC for vulnerable populations. Similar types of innovations were identified between countries - interventions seem to occur in similar settings, are directed at similar vulnerable groups, and use comparable funding sources. This suggests that different countries may be struggling with common access-related issues, despite their own specific contextual considerations.

### Expected target groups and community health sector focus

The population groups targeted in the reported innovations are the ones that we would expect from an equity perspective - people whose life trajectories are marked by experiences of social exclusion and poverty, facing complex healthcare and social needs which challenge their abilities to access PHC services. Governments invest in the community health sector to address the needs of vulnerable populations and this study demonstrated that this is a fertile space where innovations occur. However, our findings suggest that community health centres (CHCs) are carrying an unfair share of the burden in conducting this sort of innovative work to meet the needs of vulnerable groups. We also identified that interventions reported as being delivered outside the traditional clinical health service setting were limited, despite recognition that action must take place outside the health sector to address the wide range of social determinants of health which have an impact on health and access to PHC [5].

### Supply-side dominance

Our study also uncovered the supply-side dominance in terms of dimensions of access addressed by current innovative interventions, the latter focused on influencing determinants of access related to characteristics of healthcare organisations and systems [4]. We have demonstrated that demand-side access determinants - trying to enhance patients’ and populations’ abilities to access services - still receive little attention, despite current evidence suggesting that interventions aiming at improving access should be more patient-oriented, focused on self-management and health literacy approaches [41].

### Sustainability risk of NGOs and lack of shared responsibility

The government was reported as a major funder of the innovations with most other initiatives being funded by NGOs. NGOs’ activities often rely on insecure financial situations (e.g. non-recurrent funding) and therefore sustainability is at risk. The private sector was much less represented and the financial support provided to current innovations was usually provided by a single funder, suggesting a lack of shared community-wide responsibility to achieve equity of access to PHC.

### Poor integration of social and health determinants of access

The examples of interventions identified in this study have shown an imbalance between social and health determinants addressed to improve access to PHC; access determinants targeted remaining mainly health-focused. Equity of access to PHC requires interventions to take into account social and health determinants, the needs of patients and populations, as well as the resources available to them (supply- and demand-side determinants of access). Notwithstanding the fact that this has been recognised at the policy level and reported as a conclusion of several previous studies of access to healthcare [42–44], multisectoral collaboration (e.g. involving police, community hub and health centres) were uncommon in the innovations reported in this study, suggesting limited integration of health and social care for vulnerable populations.

### Critical perspective of the access framework

The Levesque et al. [24] framework used to support analysis was useful in identifying specific access dimensions addressed by the interventions reported in the survey and gave a structure to the work. It was particularly useful in identifying “where” most of the current activity occurs when trying to improve access to PHC for vulnerable populations (supply-side determinants of access), and to pinpoint areas which could benefit from further attention. It helped uncover the disparity between supply- and demand-side dimensions, as well as the lack of pairing of dimensions of access originating from both groups. In providing concrete examples of access interventions mapped against each of the proposed dimension of the framework, our study has strengthened the link between practice and conceptual work in this field. Wider uptake and use of the framework may allow comparison of studies in the future.

However, we encountered challenges in attempting to operationalise the framework. We had to refine the definitions provided for each dimension of access to undertake a more “fine grain” coding of the innovations in this study. Although the Levesque et al. [24] framework conceptualises access with equal importance given to organisational factors, patients’ and populations’ characteristics, and contexts (Fig. 1), what specifically bridges both sides (supply and demand) is less clear. Furthermore, social determinants of access were not as easily captured by the framework, and multilevel initiatives (micro, meso and macro) were harder to situate. Further conceptual work will help improve the applicability of the framework to the complex interconnections of the health and social structural dimensions of access in the PHC setting. Finally, even though conceptualisations, models and frameworks are now available to assist researchers in apprehending access to healthcare as a complex phenomenon, we still lack evidence on how to generate improved access for vulnerable populations in a practical, effective and meaningful way [4]: what works, for whom, under what circumstances.

### Study limitations

The findings from this study need to be considered in light of its limitations. The results are based on responses provided from survey participants (self-reported data), who were invited to report on what they believed to be the most striking components or aspects of an innovation. Therefore, some dimensions of an intervention might not have been represented in the description provided. In terms of survey design, the participants had the option of completing the survey using tick boxes or free text, depending on the question, and we recognise the potential difficulty in statistical analysis with qualitative data mixed with multiple choice options. However, all qualitative data were coded by a single experienced qualitative researcher (LR) and double coding was performed to ensure accuracy. In fact, we believe that the qualitative data were a significant contribution and helped us to identify other population groups and settings of interest, as well as components of intervention which were not as explicit through the multiple choice option compared to the free text description. Furthermore, the level of detail provided about innovations was relatively limited because of the short survey format. In addition, we do not know if the reported initiatives are effective in achieving improved access. The access framework used for analysis also needed to be piloted and refined, even though it was considered useful in identifying key access dimensions addressed by the reported innovations. The initial email database and contact list assembled was based on our existing research networks. Although we “cast a wide net” using a social media campaign to maximise our reach for innovations, a proportion of the reported innovations likely take origin from the current IMPACT research networks and might not be entirely representative of innovations worldwide to improve access to PHC. Furthermore, our findings do not provide a generalisable summary or overview about the breadth or characteristics of innovations to improve access to PHC, particularly as the majority of innovations reported from the survey in fact originate from Canada and Australia.

### Implications for research, policy and practice

Despite its limitations, this study highlights important questions that still need to be addressed in striving for equitable access to PHC for vulnerable populations: What is the optimal combination of supply- and demand-side dimensions of access? How can we decide which dimensions to target, based on what evidence? How can we make those choices taking into account particular settings? What might be the benefits or the risks of integrating: supply- and demand-side dimensions of access, social and PHC sectors, more than one access determinant and paired dimensions? More research is needed to answer these questions. In particular, there is a need for more rigorously undertaken systematic evaluations of initiatives that are developed, taking into account the particular context in which innovations are implemented and having indicators which cover the broad range of access determinants (health and social) for accurate measurement of the effects of intervention components on specific access dimensions. Future direction for research should also focus on testing the pairing of supply- and demand-side dimensions of access.

### Aiming towards multisectoral access initiatives

To have a wider impact and capture the wide range of social and health determinants of access to reach equity, we need to find ways of creating incentives for multiple social and health service sectors to be involved in the efforts directed at enhancing access to PHC. This would be a first step towards acting beyond the government funded community health sector (e.g. financing initiatives for mainstream General Practice). The private sector having been reported as a marginal source of funding for current innovative interventions considered as improving access to PHC, an opportunity exists for further development and raises the question of how to successfully engage with and incentivise funders across the public and private sectors to promote shared involvement and responsibility towards equity of access to PHC. One way forward could be to further support multisectoral collaboration to develop multifaceted interventions delivered at multiple levels [4], in different intervention settings which are not solely health-oriented. While continuing to work with those population groups who visit CHCs and General Practices, this would involve developing interventions where people live (e.g. home-based interventions), work (e.g. workplace interventions), or study (e.g. school-based intervention).

### Joining forces to address global access issues

Considering that similar access issues are experienced globally, sharing knowledge and strategies on how to improve access is likely to be useful, facilitating learning from each other’s successes and challenges. Joining forces in developing collaborative research programs and communities of interest could be a way to achieve this.

### Becoming more effective at engaging patients and populations to generate access

There is a need to demonstrate effective strategies to engage with vulnerable groups in a meaningful way - i.e. in a way that is empowering and allow them to take an active role in defining their priorities, goals and needs and reaching out to resources that can help them achieve this [45]. This could involve developing and rigorously evaluating initiatives with end-users, based on collaborative, participatory and co-design approaches. An opportunity exists to critically reflect on the potential risk of disempowerment and further vulnerabilisation of patients who are still not systematically involved as partners in identifying ways to improve access to PHC with consideration to their needs.

## Conclusions

This environmental scan was useful in identifying a wide range of innovations to improve access to PHC for vulnerable populations. It demonstrated that most of the current attempts at improving access to PHC involve supply-side determinants of access, to transform the way that health systems, organisations and services function. Efforts directed at enhancing abilities of patients and populations to access services (demand-side determinants) were much less prominent. Promising interventions aiming towards equity of access to PHC could expand to take into account social and health determinants of access, the specific needs of patients and populations, as well as resources available to them, using multifaceted, multilevel and multisectoral approaches. The effectiveness of interventions combining supply-side determinants, demand-side determinants and paired dimensions on improving access to PHC remains unknown and more evidence-based research around this topic is needed to close the equity gap and help vulnerable populations to get access to services that correspond to their needs.

## Additional file

Additional file 1: IMPACT Study Online Survey Questionnaire. Full Online Survey Questionnaire. (PDF 178 kb)

## Footnotes

1 The notion of vulnerability is conceived here as a combination of conditions, social trajectories and political contexts which make it difficult for people to gain control over their lives, take up challenges to improve their situation and achieve the full potential of their lives [46], in opposition to being solely the result of individual characteristics.
