# Supplementary material for: Equity of access to primary healthcare for vulnerable populations: the IMPACT international online survey of innovations
Source: Int J Equity Health. 2016 Apr 12;15:64. doi: 10.1186/s12939-016-0351-7 (PMC4828803; doi:10.1186/s12939-016-0351-7)
Supplement: Additional file 1: — IMPACT Study Online Survey Questionnaire. Full Online Survey Questionnaire. (PDF 178 kb) [file 12939_2016_351_MOESM1_ESM.pdf]

English ▼

Survey introduction

Welcome to the IMPACT survey

You can complete this survey either in English or in French by selecting your preferred language at the top right-hand corner of the screen.

Vous pouvez compléter ce sondage en français ou en anglais en choisissant votre langue de préférence dans l'onglet de sélection qui se trouve au haut de la page à droite.

Have you previously completed this survey?

- YES
- NO

IMPACT is an Australian-Canadian collaborative research program that aims to improve access to primary health care for vulnerable populations.

Our team of experienced international researchers from 15 universities in Australia, Canada, Switzerland, the US and the UK is led by Professor Jeannie Haggerty and Professor Grant Russell.

We invite you to complete this brief **5-minute** survey to tell us about an initiative that you are aware of which is at the cutting edge of change in improving access to primary health care for vulnerable populations.

Our work is funded by the *Australian Primary Health Care Research Institute* and the *Canadian Institutes of Health Research* with the *Fonds de recherche du Québec – Santé*.

More information about our project can be found in the plain language statement.

Make an **IMPACT** and click **NEXT** to continue.

Survey questions

Can you think of an example of an innovation:

A program or a service,
an approach or a model of care,
that really makes a difference
in the way that it helps vulnerable people
to get access to primary health care.

What is its name/what is it called?

- ○ Enter name
- ○ There is no example of innovation that I can think of

Choose one of the following options.

- ○ I can refer you to someone who could tell you more about this topic
- ○ I would like to exit this survey

Please provide details below.

- ☐ Enter name:
- ☐ Enter email address:

Where is it located?

Choose the country from the scroll-down list:

Enter the state or province:

Enter the name of the city:

In what setting(s) is it delivered?

Tick all boxes that apply.

- [ ] At the home
- [ ] Community health centre
- [ ] General practice/Family medicine group
- [ ] Hospital
- [ ] Mobile clinic/Outreach
- [ ] Non-governmental organisation (NGO)
- [ ] Online
- [ ] Over the telephone
- [ ] Other: please describe

Who is it designed to help?

- ○ No particular group is targeted
- ○ I would like to select the target group(s) among a list of options
- ○ I would like to describe it in free text

Tick all boxes that apply.

- [ ] Low income individuals/families
- [ ] Homeless people
- [ ] Refugees
- [ ] Culturally and Linguistically Diverse (CALD) communities
- [ ] Indigenous peoples

- ☐ Children/adolescents
- ☐ Lesbian/Gay/Bisexual/Transgender/Intersex (LGBTI) people
- ☐ Victims of violence/abuse
- ☐ People living with chronic diseases
- ☐ People living with a mental health illness and their carers
- ☐ Drug users
- ☐ People living with a disability
- ☐ Elderly people
- ☐ Other: please describe

Please describe in a few words or list all target groups that apply.

What does it do and how is it innovative?

Please describe in a few words or sentences.

How is it financed or paid for?

Tick all boxes that apply.

- ☐ Government
- ☐ Not-for-profit or charity organisation
- ☐ Private sector
- ☐ User payment
- ☐ I don’t know
- ☐ Other: please describe

How do you know about it?

Tick all boxes that apply.

- ☐ From a colleague
- ☐ I know someone who used it
- ☐ I have used it myself
- ☐ I participate/was involved in delivering this program or service
- ☐ I am/was involved in its design, implementation or evaluation
- ☐ Other: please describe

How could we find out more about this innovation?

Tick all boxes that apply and fill out the details.

- ☐ I could tell you more about it
  Enter your email address:
- ☐ A Website that we should look at
  Paste the link:
- ☐ A report that we should access
  If online, paste the link. If not, enter name.
- ☐ A person who we could talk to
- ☐ I don’t know

Please provide details below.

- ☐ Enter name:
- ☐ Enter email address:

Would you like to direct us to anyone else who might know about this

topic?

- YES
- NO

Please provide details below.

- Enter name:
- Enter email address:

ABOUT YOU...

We would like to ask a few questions about you so that we know who contributed to our survey.

Click NEXT to continue.

ABOUT YOU...

Where do you live?

Choose the country from the scroll-down list:

Enter the state or province:

Enter the name of the city:

Enter the postcode:

ABOUT YOU...

Are you:

- Male
- Female
- Other
- Rather not say

ABOUT YOU...

What age group are you in?

- Under 18
- 18-25
- 26-34
- 35-54
- 55-64
- 65 or over

ABOUT YOU...

What is the highest qualification that you have completed?

- Primary/Elementary school
- Secondary school/High school
- Certificate/diploma
- Undergraduate degree
- Postgraduate degree

ABOUT YOU...

Which role(s) best describe(s) your current area of work?

Tick all boxes that apply.

- ☐ I am not in paid work
- ☐ Family physician/General practitioner
- ☐ Manager of primary care services
- ☐ Nurse (e.g. registered nurse clinician, nurse practitioner, primary care nurse)
- ☐ Political or governmental role
- ☐ Researcher
- ☐ Social worker
- ☐ Student
- ☐ Volunteer worker
- ☐ Other health care provider: please specify ______
- ☐ Other: please describe ______

ABOUT YOU...

How did you learn about this survey?

Tick all boxes that apply.

- ☐ Email
- ☐ Twitter
- ☐ Facebook
- ☐ Linkedin
- ☐ Blog
- ☐ Website
- ☐ Word of mouth
- ☐ Other: please specify ______

THANK YOU for your time in completing our survey!

Can we contact you to talk more about this topic, if needed?

- ○ YES
- ○ NO

Please provide your contact details below.

- ☐ Name: __________
- ☐ Email address: __________
- ☐ Telephone number (including country and area code): __________

Click NEXT to finish the survey.
